# Managing African animal trypanosomiasis in Benin: Knowledge, attitudes, and practices of cattle owners in the West Atacora zone

**DOI:** 10.14202/vetworld.2025.1508-1516

**Published:** 2025-06-15

**Authors:** Yao Akpo, Aretas B. N. Tonouhewa, Traore Alkoiret, Marc T. Kpodekon

**Affiliations:** 1 Laboratory of Animal Ecology, Health and Production (LESPA), University of Parakou, Parakou, Bénin; 2 Research Unit on Communicable Diseases (URMAT), Polytechnic School of Abomey-Calavi, University of Abomey-Calavi, Abomey-Calavi, Bénin

**Keywords:** African animal trypanosomiasis, Benin, cattle farmers, knowledge-attitudes-practices, trypanocide resistance, tsetse fly control

## Abstract

**Background and Aim:**

African animal trypanosomiasis (AAT), transmitted by tsetse flies, severely constrains livestock productivity in sub-Saharan Africa. In Benin, limited governmental control initiatives and widespread drug misuse have raised concerns about emerging trypanocidal resistance. This study aimed to assess the knowledge, attitudes, and practices of cattle farmers in the Atacora and Donga departments of northern Benin and identify behaviors contributing to the persistence and drug resistance of AAT.

**Materials and Methods:**

A cross-sectional survey was conducted from September to December 2024 among 201 cattle farmers selected through stratified random sampling across five districts. Data were collected using a semi-structured, pre-tested questionnaire and analyzed with R software employing descriptive statistics and comparative tests (χ^2^, Mann–Whitney U, and Kruskal–Wallis).

**Results:**

Awareness of tsetse flies and their role in AAT transmission was high (84%), but only 24% recognized insecticide-based vector control as an effective method. The predominant control strategy involved trypanocides – mainly diminazene aceturate (81%) and isometamidium chloride (71%) – with 99% of participants administering these drugs. Notably, 42% sourced trypanocides from illicit markets, and 22% practiced self-medication. Most farmers (56%) treated their cattle twice yearly, yet 65% failed to observe withdrawal periods, and 59% reported therapeutic failures. Only 12% had received formal training in AAT management.

**Conclusion:**

Despite high disease awareness, poor adherence to recommended control practices and the prevalent misuse of trypanocides, particularly through informal markets, pose serious threats to sustainable AAT management. There is an urgent need for integrated risk communication and policy-driven interventions promoting responsible drug use and vector control in northern Benin.

## INTRODUCTION

African animal trypanosomiasis (AAT) is a parasitic disease caused by various protozoan species of the genus *Trypanosoma*, transmitted to livestock by tsetse flies. In sub-Saharan Africa, AAT significantly undermines livestock productivity in regions where the tsetse fly, the principal vector, is prevalent [1, 2]. It is estimated that over 50 million cattle, sheep, and goats across 34 African countries are at risk of AAT, resulting in annual economic losses of several billion dollars [3, 4]. Despite the deployment of vector control strategies in endemic regions, the treatment of animals using trypanocides remains the primary method of disease management in Africa. However, no new trypanocidal drugs have been commercially introduced in the past five decades. Moreover, the frequent and inappropriate use of existing molecules has accelerated the development of trypanosome resistance to these treatments [5, 6].

In Benin, AAT constitutes the most significant constraint on livestock productivity, particularly in areas where the tsetse fly is endemic. Doko *et al*. [7] and Farougou *et al*. [8] have reported variable prevalence rates depending on geographical location, with infections predominantly caused by *Trypanosoma vivax* and *Trypanosoma congolense*. According to Soha *et al*. [9], AAT is markedly more prevalent during the rainy season than the dry season, with an overall prevalence of 27.6%, as determined by the buffy coat technique in samples from several districts. In the northwestern region, Farougou *et al*. [8] observed that the disease is substantially less prevalent in sheep compared to cattle, with a reported prevalence of 6.02%. To combat AAT, farmers predominantly rely on trypanocides available through local markets. However, recurring treatment failures in recent years have raised concerns about emerging drug resistance, suggesting the onset of chemoresistant trypanosome strains in the region. This pattern aligns with similar reports from neighboring West African countries, including Togo and Burkina Faso [10–12].

Although numerous studies across sub-Saharan Africa have documented the epidemiology of AAT, most focus primarily on disease prevalence and vector distribution without adequately addressing the socio-behavioral dimensions of disease management. In Benin, while the ecological presence of *Trypanosoma* species and their tsetse fly vectors is well established, there remains a paucity of systematic investigations into the knowledge, attitudes, and practices (KAP) of livestock farmers concerning AAT prevention and control. Furthermore, the widespread reliance on trypanocides, coupled with increasing reports of therapeutic failure, raises serious concerns about the emergence of chemoresistance. However, there is limited empirical evidence on how farmers’ treatment behaviors – particularly unregulated access to drugs, self-medication practices, and disregard for withdrawal periods – may contribute to resistance development. Few studies have quantified these practices or assessed their implications for sustainable disease control strategies in Benin. In addition, the lack of training and formal veterinary support in remote rural regions further exacerbates the risk of inappropriate trypanocide use. This gap in contextual, farmer-level data hinders the design of effective, locally adapted AAT management programs.

This study aimed to evaluate the knowledge, attitudes, and practices of cattle owners regarding AAT in the Atacora and Donga departments of northern Benin. Specifically, the objectives were to (i) assess farmers’ awareness of AAT transmission, symptoms, and control strategies; (ii) investigate prevailing attitudes toward trypanocide and insecticide use; (iii) document common treatment behaviors, including drug sourcing and administration patterns; and (iv) identify practices potentially contributing to trypanocide resistance. The ultimate goal is to generate evidence that can inform risk communication campaigns, policy interventions, and integrated disease control strategies that align with the local realities of livestock farming communities in Benin.

## MATERIALS AND METHODS

### Ethical approval and Informed consent

This study involved no experimental procedures on animals and therefore did not require formal ethical approval from a local ethics committee. Verbal informed consent was obtained from each participating farmer before their inclusion in the survey.

### Study period and location

**Figure 1:**
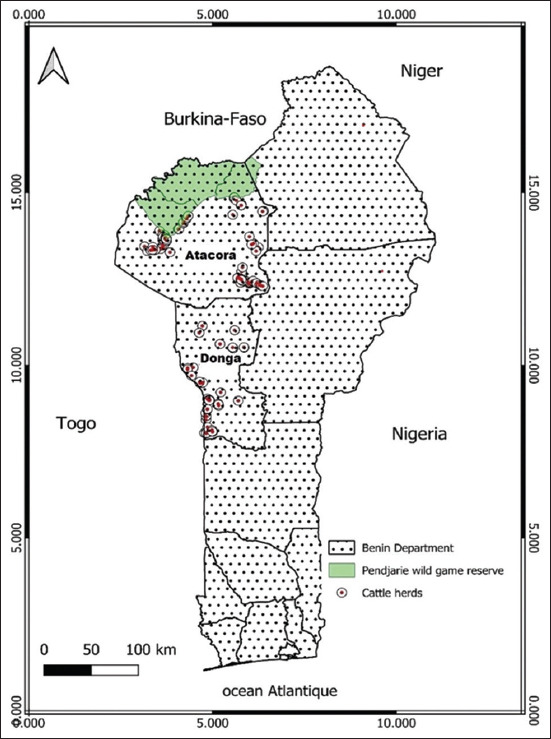
Study area map [Source: The map was generated using ArcGIS 10.8, Environmental System Research Institute (ESRI), USA].

The study was conducted from September to November 2024. The study was conducted in the Republic of Benin, a West African country (Figure 1). A cross-sectional, descriptive survey utilizing stratified random sampling was conducted among cattle farmers in five districts: Bassila, Djougou, Kerou, Pehunco, and Tanguieta, located in the Atakora and Donga departments in the northeastern region of the country. These districts span an area of approximately 20,737 km². The prevailing climate in the study region is of the Sudanian type, and the dominant vegetation is savanna woodland. Several wildlife reserves and protected forest areas are located within this zone, including the Pendjari Wildlife Reserve – bordering Tanguieta, Kerou, and Pehunco – and the Wari-Maro classified forest in Bassila. These habitats offer favorable ecological conditions for the proliferation of tsetse flies, the vectors responsible for the transmission of AAT.

### Study design and sampling strategy

A cross-sectional survey was conducted to evaluate cattle farmers’ KAP related to AAT, as well as their usage patterns of trypanocidal drugs in northeastern Benin. The minimum required sample size was determined using Thrusfield’s formula [13], based on an assumed 90% prevalence of adequate knowledge regarding AAT clinical signs [14], with a 5% margin of error and a 95% confidence interval. This calculation yielded a minimum target sample of 139 farmers. Ultimately, 201 farmers were surveyed. In each district, five villages were randomly selected using a probability-proportional-to-size sampling approach. Within these villages, cattle owners were identified in collaboration with local livestock farmer associations to ensure population representativeness. The inclusion criteria stipulated that participants must be at least 18 years old and have actively practiced cattle farming in the selected districts for a minimum of 2 consecutive years, to ensure familiarity with local disease conditions and management practices.

### Questionnaire development and data collection

A semi-structured questionnaire was rigorously developed, drawing upon validated instruments from prior KAP studies conducted in Gambia, Mali, Burkina Faso, and Guinea [14, 15]. Before full-scale deployment, the questionnaire was pilot-tested on a small group of cattle farmers outside the study area to assess clarity, cultural relevance, and reliability. The finalized questionnaire comprised four main sections: (1) Sociodemographic characteristics; (2) knowledge about AAT and its control strategies; (3) attitudes toward AAT prevention and control; and (4) practical approaches to disease management. Data collection was conducted using KoboCollect, a mobile data collection application that enables real-time entry through trained enumerators. Enumerators received standardized training sessions to reduce inter-observer variability and ensure consistency in data gathering.

### Statistical analysis

The collected data were exported from Kobo-Collect into Microsoft Excel 2016 for initial screening, including consistency checks and recoding where necessary. The cleaned dataset was subsequently imported into R software (version 4.0.5) for comprehensive statistical analysis. Descriptive statistics, including means and frequencies, were calculated for key variables of interest. For all analyses, missing values were excluded from denominators to ensure accurate frequency estimations. Inferential analyses involved both parametric (Chi-square test) and non-parametric methods (Mann–Whitney U and Kruskal–Wallis tests) to evaluate differences in categorical and continuous variables, respectively, between participants from Atak-ora and Donga. Statistical significance was established at a threshold of α = 0.05.

## RESULTS

### Sociodemographic characteristics of farmers

Table 1 presents sociodemographic characteristics of cattle owners. Among the 201 participants surveyed across the five municipalities, the majority of farmers (65%, n = 127) were aged between 35 and 60 years. The sample was overwhelmingly male (97.5%, n = 195), with only a small proportion of women (2.5%) engaged in cattle farming as a primary activity, reflecting the male-dominated nature of livestock production in the region. A substantial proportion of participants (74%, n = 145) had no formal education, though more than half (59%) reported over 10 years of experience in cattle farming.

**Table 1:** Sociodemographic characteristics of the participants.

Variables	n (%)
Department	
Donga	78 (38.8)
Atacora	122 (62.2)
Sex	
Female	5 (2.5)
Male	195 (97.5)
Age	
35–60 years old	127 (65)
18–35 years old	52 (27)
Over 60 years old	17 (8.7)
Professional experience	
1–5 years	11 (5.47)
5–10 years	69 (34.3)
Less than 1 year	1 (0.49)
More than 10 years	118 (59)
Education	
Primary education	37 (19)
Secondary education	15 (7.6)
No formal education	145 (74)
Livestock breeding as the main activity	
No	17 (8.6)
Yes	181 (91)
Animal species	
Local cattle breeds	193 (97)
Exotic cattle breeds	5 (2.5)
Crossbred cattle	4 (2.0)
Goats and sheep	144 (72)
Poultry	50 (25)
Grazing mode	
Communal	170 (86)
Inter-communal	85 (43)
Departmental	21 (11)
Transhumance practice	
No	119 (64)
Yes	68 (36)
Endemic diseases	
Bacterial diseases (mastitis, diarrhea, etc.)	172 (86)
Dermatoses	89 (45)
Bovine trypanosomosis	192 (96)
Internal parasitosis	51 (26)
Tick and tick-borne diseases	78 (39)
Foot and mouth diseases	96 (48)

Regarding livestock breeds, 97% (n = 193) of far-mers reared only local taurine breeds – such as Borgou, Somba, zebu, and their crosses – while a minority (2.5%, n = 5) kept exotic Girolando cattle. A significant number of farmers engaged in mixed-species farming: 72% kept small ruminants (n = 144), 72% kept pigs (n = 144), and 25% kept poultr`y (n = 50). Sedentary livestock production was the dominant system, practiced by 64% (n = 119) of participants, while the remaining 36% (n = 68) practiced transhumance, particularly during the dry season, beginning in December when pasture resources are limited.

Health challenges were frequently reported, with trypanosomosis being the most prevalent condition (96%, n = 192), followed by diarrhea and mastitis (86%, n = 172), foot-and-mouth disease (23.8%, n = 48), and dermatological conditions (45%, n = 45).

### Farmers’ knowledge of AAT

As presented in Table 2, the majority of participants (84%, n = 164) demonstrated the ability to identify *Glossina* (tsetse flies) based on their wing morphology and irritating behavior, with farmers in the Donga department exhibiting significantly greater knowledge (99%, n = 76/77) than those in Atacora (74%, n = 88/123; p < 0.001). In local dialects, tsetse flies were referred to as *Faa tchonka* (Waarma) and *Boubel goorou* (Peuhl), both meaning “swamp fly” in English.

**Table 2:** Cattle owners’ knowledge of tsetse flies and AAT.

Variables	Atacora N = 123 n (%)	Donga N = 78 n (%)	Total N = 201 n (%)	p-value
Knowledge of the tsetse fly				<0.001
No	31 (26)	1 (1.3)	32 (16)	<0.001
Yes	88 (74)	76 (99)	164 (84)	<0.002
In which area are tsetse flies most abundant?				<0.06
In pastures with many trees (forests)	36 (30)	21 (27)	57 (28.3)	0.7
In areas where wild animals are present,	46 (38)	33 (43)	79 (37.3)	0.5
In pastures near rivers and lowlands	113 (93)	67 (87)	170 (79)	0.13
I do not know	1 (0.8)	2 (2.6)	3 (1.4)	0.6
In which areas are cattle most bitten by tsetse flies?				0.012
In pastures with many trees (forests)	70 (57)	61 (79)	131 (66)	0.002
In areas where wild animals are present	45 (37)	24 (31)	69 (35)	0.4
In pastures near rivers and lowlands	96 (79)	42 (55)	138 (69)	<0.001
I do not know	4 (3.3)	3 (3.9)	7 (3.5)	>0.9
What time of year are tsetse flies most abundant?				<0.001
I do not know	3 (2.5)	4 (5.6)	7 (3.6)	0.06
Rainy season	109 (89)	41 (58)	150 (78)	0.04
Dry season	1 (0.8)	16 (23)	17 (8.8)	<0.001
All year round	9 (7.4)	10 (14)	19 (9.8)	0.003
What diseases do tsetse flies transmit to cattle?				0.4
Trypanosomoses	105 (85.3)	66 (84.6)	171 (85)	0.7
I do not know	18 (14.7)	12 (15.4)	30 (14.9)	0.5
Symptoms of trypanosomosis				>0.05
Hair loss	105 (86)	68 (87)	173 (87)	0.8
Weight loss/emaciation	83 (68)	28 (36)	111 (56)	<0.001
Low milk production	71 (58)	48 (62)	119 (60)	0.6
Nervous symptoms such as walking in circles	26 (21)	13 (17)	39 (20)	0.4
Tearing	67 (55)	41 (53)	108 (54)	0.7
Inappetence	43 (35)	25 (32)	68 (34)	0.6
Constipation	3 (2.5)	3 (3.8)	6 (3.0)	0.7
Swollen lymph nodes	14 (11)	3 (3.8)	17 (8.5)	0.059
Hypersalivation	3 (2.5)	1 (1.3)	4 (2.0)	>0.9
Diarrhea	7 (5.7)	22 (28)	29 (15)	<0.001
What is the best way to combat animal trypanosomosis?				>0.6
Use insecticides	36 (30)	12 (15)	48 (24)	0.023
Use antibiotics	46 (38)	30 (38)	76 (38)	>0.9
Burning plants to repel tsetse flies	54 (44)	24 (31)	78 (39)	0.056
Use trypanocides	101 (83)	75 (96)	176 (88)	0.005
Use of medicinal plants to treat animals	14 (11)	9 (12)	23 (12)	>0.9
I do not know	1 (0.8)	0 (0)	1 (0.5)	>0.9
Have you received training in managing animal trypanosomosis management?				0.014
No	109 (92)	57 (80)	166 (88)	0.03
Yes	9 (7.6)	14 (20)	23 (12)	0.001

AAT= African animal trypanosomiasis

A significant proportion of farmers identified high-risk zones – such as wetlands, rivers, and dense vegetation – as common grazing areas associated with high tsetse fly abundance (79.6%, n = 160/201). In addition, 84.5% (n = 170/201) correctly recognized that tsetse flies transmit trypanosomiasis to animals, commonly referred to as *massah* in Fulani.

Farmers were also able to identify key clinical symptoms associated with AAT, including hair loss (87%, n = 173), emaciation or anemia (56%, n = 111), reduced milk production (60%, n = 119), and lacrimation (54%, n = 108). The most widely recognized control strategy was the use of trypanocides (88%, n = 176), while only 24% (n = 48) were aware of the effectiveness of insecticides in controlling tsetse fly populations.

Furthermore, 39% (n = 78) of farmers reported using fumigated medicinal plants as repellents around stabling areas to deter tsetse flies. Notably, only 12% of participants had ever received formal training on AAT prevention or management.

### Farmers’ attitudes toward AAT control methods

A relatively small proportion of farmers (15%, n = 29) believed that insecticides constituted an effective strategy for preventing or managing AAT. In contrast, an overwhelming majority (96%, n = 191) expressed greater confidence in trypanocidal drugs. When asked about the perceived drivers of rising AAT incidence, most participants attributed the increase to the presence of tsetse flies in the region, while only 16% (n = 31) acknowledged the possibility of drug resistance (Table 3).

**Table 3:** Farmer attitudes toward AATand control strategies.

Variables/Question	Atacora N = 123 n (%)	Donga N = 78 n (%)	Total N = 201 n (%)	p-value
What explains the increase in trypanosomosis case in your farm?				<0.002
Presence of tsetse	115 (94)	58 (74)	173 (87)	<0.001
Lack of information on means of prevention and control methods	47 (39)	46 (59)	93 (47)	0.005
Lack of veterinarians on the ground to support breeders in the fight	26 (21)	29 (37)	55 (28)	0.014
Treatments for the disease are no longer effective	16 (13)	15 (19)	31 (16)	0.2
I do not know	2 (1.6)	1 (1.3)	3 (1.5)	>0.9
What strategy is the most effective for fighting animal trypanosomosis?				
Use of medicinal plants	33 (27)	32 (42)	65 (33)	0.034
Use of trypanocides	118 (97)	73 (95)	191 (96)	0.7
Using insecticides to repel flies	15 (12)	14 (18)	29 (15	0.3

AAT=African animal trypanosomiasis

### Management practices related to AAT control

Farmers in the study area employed a variety of methods to manage AAT in their herds. These included the use of insecticides (34%, n = 67), the application of waste motor oil on cattle to deter ectoparasites (36%, n = 72), and burning medicinal plants near stables (43%, n = 85). Chemical trypanocides were overwhelmingly used (99%, n = 199), with many farmers also employing ethnoveterinary approaches using medicinal plants (33%, n = 65).

The primary trypanocides reported were diminazene aceturate (81%, n = 162) and isometa- midium chloride (71%, n = 142). These products were most commonly procured from private-sector animal health workers (71%, n = 140), though 42% (n = 83) of participants admitted purchasing drugs from unregulated or illicit sources, including livestock markets and fellow farmers. Drug administration was typically performed by private veterinarians or para-veterinarians (76%, n = 145), but self-administration by farmers was also notable (22%, n = 41).

Regarding treatment frequency, more than half (56%, n = 112) treated their animals twice per year, while 22% and 4% conducted treatments three and four times annually, respectively. The average cost of treatment per cow was 750 West African CFA francs (XOF, ~$1.20), with regional variation – Atacora (700 XOF) and Donga (1,200 XOF). Notably, 65% (n = 130) of farmers did not comply with recommended withdrawal periods for milk and meat post-treatment. Moreover, 59% (n = 117) reported experiencing at least one therapeutic failure after trypanocide administration (Table 4).

**Table 4:** Farmer practices related to AAT and control strategies.

Variables	Atacora N = 123 n (%)	Donga N = 78 n (%)	Total N = 202 n (%)	p-value
Have you ever tried to stop tsetse flies from biting your cattle?				<0.001
No	49 (40)	12 (16)	61 (31)	<0.001
Yes	72 (60)	64 (84)	136 (69)	0.002
To prevent tsetse flies from biting your cattle, what control method have you used?				>0.05
Burning medicinal plants near herds to repel tsetse flies	51 (42)	34 (44)	85 (43)	0.7
Apply motor oil to the body of animals.	50 (41)	22 (29)	72 (36)	0.076
Spray animals with commercially available insecticides.	39 (32)	28 (36	67 (34%)	0.5
None	6 (4.9)	4 (5.2)	10 (5.0)	>0.9
What trypanocide have you used to treat cattle in the past 3 months?				<0.04
Isometamidium chloride (Red bag)	85 (70)	57 (73)	142 (71)	0.7
Diminazene Aceturate (Yellow bag)	90 (74)	72 (92)	162 (81)	0.002
Where do you buy trypanocides?				>0.05
Among private animal health workers	66 (55)	37 (49)	53	0.5
At the veterinary pharmacy	84 (69)	56 (75)	140 (71)	0.4
Among government animal health workers	36 (30)	1 (1.3)	37 (19)	<0.001
At other farmers	4 (3.3)	2 (2.7)	6 (3.1)	>0.9
The Black Market	59 (49)	24 (32)	83 (42)	0.021
Who administers the medicine to the animals?				0.002
A para-veterinarian	0 (0)	1 (1.4)	1 (0.5)	0.07
A veterinarian	81 (69)	64 (86)	145 (76)	0.003
Another farmer	3 (2.5)	2 (2.7)	5 (2.6)	0.06
Yourself	34 (29)	7 (9.5)	41 (21)	0.001
How often do you perform the treatment?				<0.001
Twice a year	74 (61)	38 (49)	112 (56)	0.003
Three times a year,	15 (12)	29 (37)	44 (22)	0.002
Once a year, every 4 weeks	32 (26)	4 (5.1)	36 (18)	0.001
Four times a year	1 (0.8)	7 (9.0)	8 (4.0)	0.005
Do you respect the waiting period for milk and meat after trypanocide treatment?				<0.001
No	103 (84)	27 (35)	130 (65)	<0.001
Yes	19 (16)	51 (65)	70 (35)	<0.001
Does it often happen that your animals are not cured after treatment?				0.4
No	46 (38)	34 (44)	80 (41)	0.05
Yes	74 (62)	43 (56)	117 (59)	0.04
Do you think that trypanosomoses in animals can be effectively controlled?				<0.001
No	50 (41)	13 (17)	63 (32)	<0.001
Yes	72 (59)	65 (83)	137 (69)	0.002

AAT=African animal trypanosomiasis

## DISCUSSION

### Prevalence and socioeconomic impacts of AAT in Northern Benin

AAT is highly endemic in Benin, particularly in the northern regions, where its prevalence is most pronounced. In these areas, cattle farmers report significant annual losses in both milk and meat production due to AAT-related morbidity [7–9]. A study conducted in the Atacora-Ouest agroecological zone confirmed that while cattle ownership is common among rural and peri-urban households, men are predominantly engaged in livestock rearing, whereas women are typically involved in milk processing and marketing [8]. This gendered division of labor reflects broader patterns observed throughout West Africa, where men manage large herds and women handle secondary value-chain activities [16, 17].

Demographically, the majority of surveyed farmers were over 35 years old (73%) and lacked formal education (74%), although 59% had more than a decade of livestock farming experience. These characteristics align with observations by Serem *et al*. [18], who noted that younger and more educated individuals tend to migrate toward urban centers in search of salaried employment, leaving older populations to sustain agricultural activities in rural settings.

### High awareness of tsetse flies and AAT transmission

The survey revealed high levels of awareness among farmers regarding tsetse flies and their role in transmitting AAT. Over 80% of participants could distinguish tsetse flies from other insects by their size and wing morphology. Most were also aware of the ecological conditions conducive to tsetse proliferation – namely, areas near rivers, wetlands, and dense vegetation. The presence of tsetse fly habitats in and around forested reserves, such as the Pendjari National Park (bordering Tanguiéta, Kerou, and Pehunco) and the Wari-Maro classified forest in Bassila, likely reinforces this awareness. These zones are also inhabited by wildlife species known to serve as trypanosome reservoirs, including *Adenota kob* (antelopes), *Phacochoerus aethiopicus* (warthogs), and lions, which have previously been documented as infected hosts [19].

Nearly 70% of livestock owners recognized that the likelihood of tsetse bites increased in wooded pastures, especially near wildlife reserves. These findings are consistent with those of Grace *et al*. [14], who reported similar levels of vector-related knowledge in Burkina Faso, Mali, and Guinea, and Kargbo *et al*. [15], who found even higher awareness (94.5%) in the Gambia.

### Vector and pathogen dynamics in Northern Benin

Dehoux and Hounxou-Ve [20] have confirmed the widespread presence of tsetse fly species in northern Benin, notably *Glossina tachinoides*, *Glossina palpalis gambiensis*, and *Glossina morsitans submorsitans*, all competent vectors of AAT. The risk of disease transmission intensifies when such vectors coexist with wildlife reservoirs, as is the case in this region [6]. The seasonal spike in AAT cases during the rainy season – despite sufficient forage availability – can be attributed to increased tsetse fly activity at that time, a phenomenon corroborated by Soha *et al*. [9]. Consequently, farmers often associate the onset of clinical signs – such as emaciation, reduced milk yield, alopecia, and lacrimation – with tsetse exposure and promptly seek treatment from veterinary personnel. Similar knowledge patterns have been reported among Maasai herders in Kenya [18].

### Attitudes and misconceptions regarding AAT control

Despite widespread knowledge of the disease and its vectors, attitudes toward integrated AAT control remain suboptimal. Only 34% of farmers reported using insecticides for tsetse control, while most expressed greater confidence in curative treatment with trypanocides. These results are in line with those obtained in the Kenyan region of Arabuko Sokoke, where only 30% of farmers used vector control strategies [18]. Such preferences suggest a disconnect between awareness and preventive behavior, highlighting the need for targeted health education programs that promote integrated AAT management by combining chemical vector control (e.g., insecticides or traps) with the judicious use of trypanocides.

### Treatment practices and risk of drug resistance

Diminazene aceturate was the most commonly used trypanocide (81%), followed by isometamidium chloride (71%), despite the latter being less expensive on the local market. This preference is likely due to the stronger curative efficacy of diminazene acet-urate [6], a trend also observed in other endemic countries [10, 11]. However, the near-universal and often unsupervised use of these molecules raises serious concerns regarding the development of drug resistance. In particular, 21% of participants reported practicing self-medication, and 41% admitted sourcing trypanocides from informal markets, where drug qua-lity cannot be assured.

These findings suggest a high risk of resistance emergence due to non-compliance with veterinary guidelines, such as disregarding drug withdrawal periods, and poor-quality drug use. This aligns with reports from other West African countries, including Senegal, Mali, and Togo, where resistance to isome-tamidium chloride and other trypanocides has already been documented [5, 12, 14, 21]. The 59% rate of therapeutic failure reported by surveyed farmers may, therefore, reflect the cumulative effects of these substandard practices.

### Potential of ethnoveterinary medicine

Farmers also reported using ethnoveterinary approaches, such as burning *Hyptis suaveolens* to repel tsetse flies and treating sick animals with plant-based preparations, including extracts from *Khaya senegalensis*. These practices, grounded in trad-itional knowledge, could offer viable alternatives or complements to chemical control methods, particularly in light of growing concerns about drug resistance. The pharmacological potential of these plants warrants further investigation to identify and isolate bioactive compounds that may serve as new anti-trypanosomal agents [22, 23].

## CONCLUSION

This study provides critical insights into the knowledge, attitudes, and practices of cattle farmers concerning AAT in the Atacora and Donga departments of northern Benin. The findings reveal that while a substantial proportion of farmers (84.5%) possess adequate knowledge of tsetse fly vectors and clinical symptoms of AAT, including hair loss, anemia, and reduced milk yield, only a minority (24%) recognize the importance of vector control through insecticides. Instead, most rely heavily on trypanocides, particularly diminazene aceturate (81%) and isometamidium chloride (71%), with widespread self-administration (22%) and procurement from unre-gulated markets (42%).

These practices pose significant risks for the emergence of trypanocide resistance, as evidenced by the high proportion (59%) of reported therapeutic failures. The disregard for withdrawal periods (65%) further raises concerns about food safety and public health. These findings underscore the urgent need for targeted risk communication campaigns and farmer training programs that promote integrated control strategies, combining judicious drug use with tsetse fly management.

This research is among the first in Benin to quantitatively assess the behavioral and treatment dimensions of AAT control using a stratified, repres-entative sample across five districts. The incorporation of validated KAP instruments adapted to local contexts adds methodological rigor and cultural relevance.

The study relied on self-reported practices, which may be subject to recall or social desirability bias. In addition, the absence of laboratory confirmation of drug resistance limits the ability to draw direct cau-sal links between reported treatment failures and chemoresistance.

Further research should incorporate molecular diagnostics to characterize resistance patterns in *Trypanosoma* spp. and assess the pharmacovigilance of trypanocides in local markets. Evaluating the efficacy of plant-based ethnoveterinary interventions, such as *Khaya senegalensis* and *Hyptis suaveolens*, could offer alternative or adjunctive strategies to mitigate chemical resistance. Longitudinal studies examining the impact of community education and regulatory policies on treatment behaviors would also be valuable.

In conclusion, this study highlights the pressing need to bridge the gap between disease awareness and best management practices among livestock farmers. Strengthening veterinary extension services and fostering participatory, evidence-based control strategies are essential steps toward sustainable AAT management and improved livestock productivity in endemic regions of West Africa.

## DATA AVAILABILITY

The supplementary data can be made available from the corresponding author upon request.
